# The phenotypic characteristic observed by cardiac magnetic resonance in a PLN-R14del family

**DOI:** 10.1038/s41598-020-73359-8

**Published:** 2020-10-05

**Authors:** Xincheng Jiang, Yuanwei Xu, Jiayu Sun, Lili Wang, Xinli Guo, Yucheng Chen

**Affiliations:** 1 Department of Cardiology, West China Hospital, Sichuan University, Guoxue Xiang No. 37, Chengdu, Sichuan 610041 People’s Republic of China; 2 Department of Radiology, West China Hospital, Sichuan University, Chengdu, 610041 Sichuan China

**Keywords:** Genetics, Cardiology, Diseases, Signs and symptoms

## Abstract

Phospholamban (PLN) is an important regulator for sarcoendoplasmic reticulum (SR) calcium transport ATPase (SERCA), which uptakes Ca^2+^ to SR during the diastolic phase of cardiomyocytes to maintain intracellular calcium homeostasis. Mutations on PLN result in intracellular calcium disorder, myocardial contraction defect, and eventually heart failure and/or malignant ventricular arrhythmia. Since 2003, several kinds of PLN mutations have been identified in familial dilated cardiomyopathy (DCM) patients, illustrating a few clinical characteristics that differs from classical DCM patients. Herein, we report a large PLN-R14del family with typical clinical characteristics reported including relatively late-onset clinical symptoms, low-voltage in ECG, as well as frequent ventricular arrythmias. Moreover, members underwent cardiac magnetic resonance (CMR) examination showed a strikingly similar pattern of late gadolinium enhancement (LGE)—Sub-epicardial involvement in the left ventricular (LV) lateral wall with or without linear mid-wall enhancement in the interventricular septum. The former one can also present in younger PLN-R14del carriers despite completely normal LV structure and function. Meanwhile, T1 mapping also found significantly increased extracellular volume (ECV) in PLN-R14del carriers. These findings highlight the special role of CMR to phenotyping PLN-induced cardiomyopathy patients and distinguish them from other types of cardiomyopathy.

## Introduction

Dilated cardiomyopathy (DCM) is one of the most common causes of heart failure. An increasing proportion (35%) of DCM was claimed to involve genetic causes^1^. The pathogenic mutations were discovered in multiple areas including sarcomere, cytoskeleton, nuclear envelope, and ion channels, etc.

The mutations in PLN implicate a special mechanism to induce DCM by influencing the intracellular calcium homeostasis of cardiomyocytes. The systole of cardiomyocytes is triggered by the rhythm of calcium sparks released by RyR2, and the diastole is delicately regulated by SERCA, which uptakes intracellular calcium back to sarcoplasmic reticulum^2^. Dynamic control of SERCA activity relies on the phosphorylation and dephosphorylation, as well as several conformational changes of phospholamban (PLN), a 52-acid protein^3,4^. PLN attaches SERCA to form a complex to inhibit the ability of SERCA, and the inhibition is mainly released by the phosphorylation by PKA.

The mutations occurred in PLN gene may not only influence the ability of PLN to inhibit SERCA but also impact the way PKA works to regulate PLN, and therefore blunt the effect of β-adrenergic receptor. Moreover, certain types of PLN mutations can form a kind of cardiotoxic “PLN” which may exert a dominant negative effect on remaining normal PLNs and their way to interact with PKA and SERCA^5^. The region between 9 and 14 seems to be the hotspot of pathogenic mutations because it contains the loci for PKA to recognize PLN. PLN-14Rdel is a common mutation that occurs in the 14 arginine. PLN with the 14 arginine deleted retains partial function of original PLN, but it exerts a super-inhibition on SERCA, as it not only fails to be phosphorylated by PKA , but also interferes the normal PLN function in heterozygous patients^6^. Chronic inhibition of SERCA elevates the intracellular Ca^2+^, blunts the calcium sparks, and ultimately leads to cardiomyopathy, heart failure, and lethal ventricular arrythmia.

Other than typical characteristics of DCM like the enlargement of LV volume and the reduction of LV ejection function (LVEF), more specific clinical characteristics have been observed in PLN-R14del patients, including a female dominant morbidity, late-onset heart failure symptoms together with early-onset sudden death risk, a low voltage of QRS and abnormal T wave mainly on anterior-lateral precordial leads, and an involvement in right ventricular and anterolateral wall of left ventricular^5,7,8^. In this study, we report the first large PLN-R14del family observed in southwestern China, with all of the mutation carriers underwent contrast-enhanced cardiac magnetic resonance imaging and Holter. Through this family of patients, we aimed to demonstrate the phenotypic characteristics on CMR and suggest the possible association between PLN mutation and phenotypes.

## Methods

The study contacted all available family members of the proband and put 9 relatives suspected to carry a pathological mutation underwent comprehensive evaluation. This study has been approved by the ethics committee of West China Hospital, and written informed consents were obtained from all participants. All research was performed in accordance with relevant guidelines/regulations.

### Clinical examination

All of the 10 members including proband went to West China hospital to receive a basic evaluation. The inquiry includes the subjective symptoms (activity chest tightness, palpitation and nocturnal dyspnea, etc.), clinical history (HF, SCD, LBBB, ICD, HTX, sustained/nonsustained VT), as well as current situation of unavailable members that died or feel not convenient to come. Pro-NT-BNP quantitation, blood routine test, 12-leads ECG and echocardiography were obtained on each individual, and the patients confirmed as PLN mutation carriers underwent a 24-h dynamic electrocardiogram (Holter). Peripheral blood was taken by experienced nurses in West China hospital and carefully centrifuged and stored in − 80 ºC.

### Genetic analysis

The genetic panel for next generation sequencing contains 117 genes reported to cause cardiomyopathy, according to OMIM (https://omim.org) and PubMed (https://www.ncbi.nlm.nih.gov/). TruSeq DNA sample preparation kit was used for the extraction and capture of DNA. The sequencing platform is Hiseq. Sanger sequencing was used for further confirmation of genetic results. Pathogenicity determination of mutation was performed following guideline recommendations^9^.

### Cardiac magnetic resonance imaging

CMR was ECG-gated and performed by a 3.0 T scanner (Magnetum Tim Trio; Erlangen, Germany), with a 32-channel cardiac phase-array receiver coil. Steady-state free precession (SSFP) cine images were acquired in consecutive short-axis views and three long-axis views (two/three/four chamber). The typical SSFP cine parameters were as follows: repetition time (TR): 3.4 ms, echo time (TE): 1.3 ms, flip angle (FA): 50°, field of view (FOV): 320–340 mm, matrix size: 256 × 144, slice thickness: 8 mm, with no gap. LGE images were acquired at 10–15 min after intravenous administration of 0.15 mmol/kg gadopentetate dimeglumine (Magnevist, Bayer Schering Pharma, Berlin, Germany) using the inversion recovery method with phase-sensitive reconstruction (PSIR) on identical short and long axis views with typical parameters as follows: TR 700 ms, TE 1.56 ms, FA 20°; matrix 256 × 144; Inversion time (TI) was individually optimized to null normal myocardial signal using a TI scout sequence. The LV structural and functional parameters were assessed with dedicated software (Qmass 7.6, Medis, Leiden, the Netherlands). Parameters including: Left Ventricular Mass (LV Mass), Left Ventricular End-Diastolic Dimension (LVEDD), Left Ventricular Ejection Fraction (LVEF), Left Ventricular End-Diastolic Volume (LVEDV), Left Ventricular End-Systolic Volume (LVESV). The borders of epicardium and endocardium was manually traced on short-axis cine images by an experienced radiologist. As for the assessment of native T1, T2 and extracellular volume (ECV), the contours were traced more conservatively, with great care to exclude blood pool, epicardial fat and trabeculations. Motion corrected modified Look-Locker inversion recovery sequence (MOLLI) was used for Native T1 measurements, with parameters as follows: TR 740 ms, TE 1.06 ms, FA 35°, bandwidth 930 Hz/pixel, initial T1 100 ms with 80 ms increments, parallel imaging factor 2, matrix 192 × 144, in plane spatial resolution 2.4 * 1.8 mm, total acquisition time 11 heart beats. The ECV was calculated as follow: ECV = (1 − HCT)  *  ([1/T1myo post − 1/T1myo pre]/[1/T1blood post − 1/T1blood pre]).

## Results

### Genotypes

Genotyping of the proband discovered a non-frameshift deletion at the loci 6q22, which is known as PLN-14Rdel. Further screening for her relatives confirmed 4 individuals carrying the same mutations—the two younger sisters (II8 and II10) and their daughters (III5 and III6).

### Clinical characteristics and phenotypes

**Figure 1:**
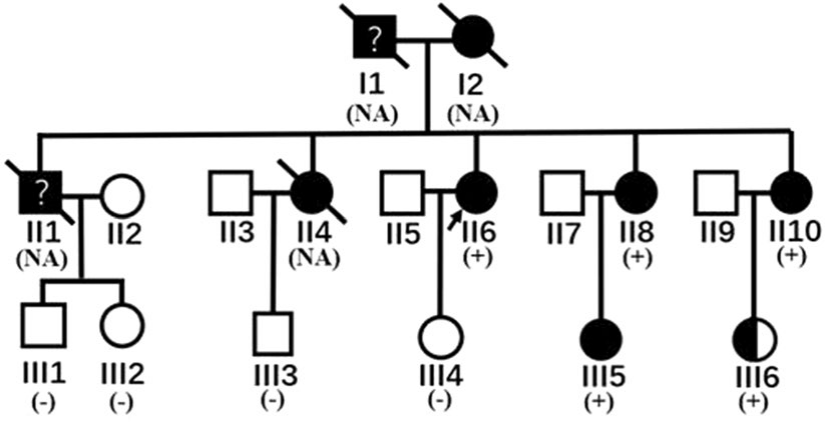
Pedigree of the family. Squares and circles represent male and female members, respectively; Slants represent dead members; arrow shows the proband; symbols filled with black indicate patients confirmed to have cardiac dysfunction as DCM; the half-filled symbol represents the member who carries the pathological mutation without significant evidence of cardiac dysfunction; the question mark represents the member whose clinical data was not available and the diagnosis was not confirmed. The members underwent a genetic testing was marked as “ + ” or “−”, and those who did not have a sequencing was marked as “NA”.

**Figure 2:**
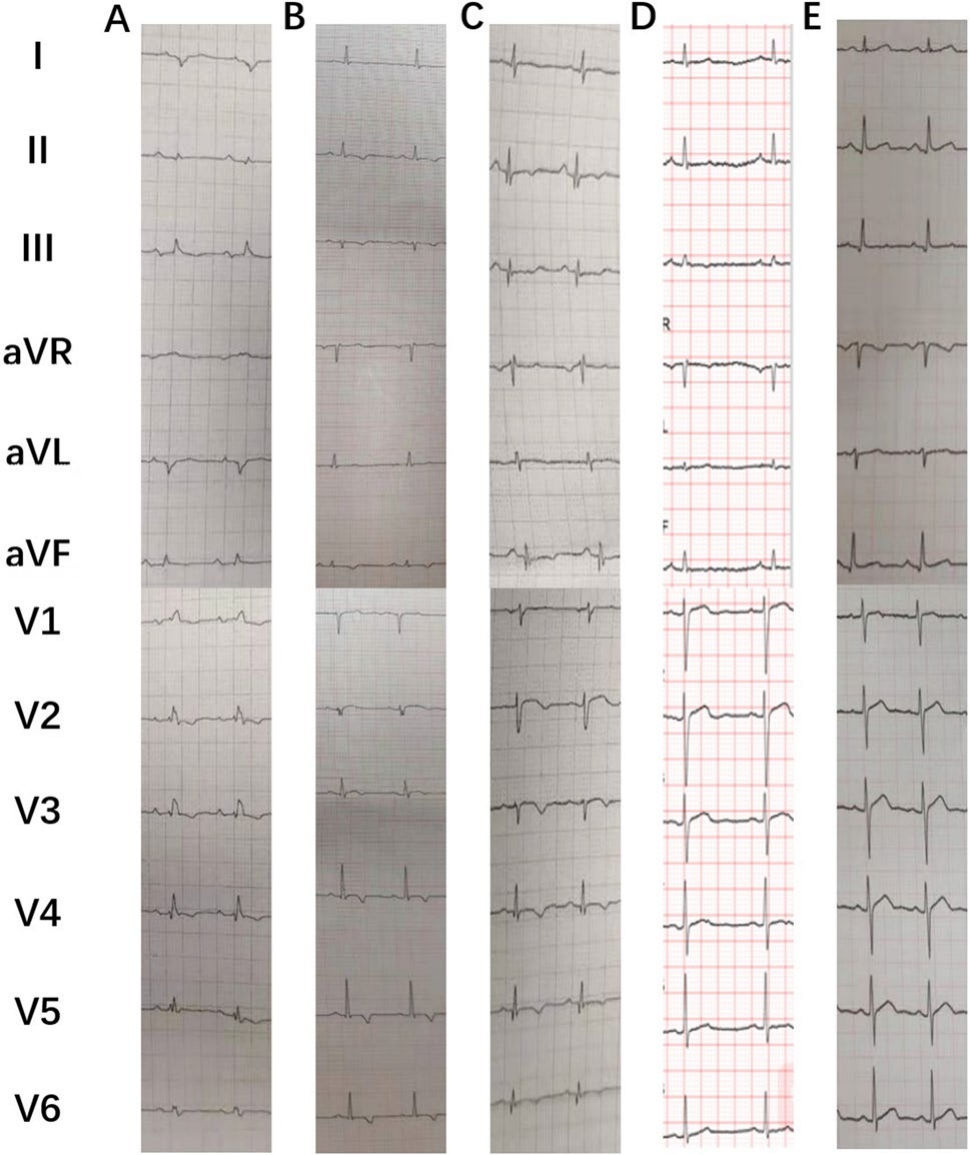
ECG of II6(A), II8(B), II10(C), III5(D), III6(E). II6(A) showed significant low amplitude of QRS in all leads, abnormal T waves in V2–V6, and complete right bundle branch block; II8(B) and II10(C) showed a similar low potential in all leads, the T waves in II, III, avF, V4–V6 are inversed in II8 and biphasic in II10, respectively; III5(D) showed relatively flat T waves in II, III, aVF; III6(E) is completely normal in ECG.

The pedigree of the PLN-14Rdel family is in Fig. 1. The proband’s elder sister, elder brother, and mother were all died of advanced heart failure at their 50 s, her father was diagnosed as myocardial infarction and died at 70 s. The proband II6 is a 60-year-old woman, who suffered from activity chest tightness for 10 years and the symptom had aggravated for recent 2 years. 12-leads ECG showed significant low amplitude of QRS in all leads, abnormal T waves in V2–V6, complete right bundle branch block, and premature ventricular contraction (PVC), with the QRS duration 130 ms, P wave 112 ms, and PR interval 190 ms (Fig. 2). Holter recording showed a ventricular escape rhythm, along with PVCs, AVCs and non-sustained ventricular/atrial tachycardia. The NT-pro-BNP progressively elevated and reached as high as 26,842 pg/ml before heart transplantation. Echocardiography showed global heart dilation and severe mitral/tricuspid regurgitation, with the LVEDD 69 mm and LVEF 40%. Beta-blocker, angiotensin-converting enzyme inhibitor, diuretic and digoxin was administered to prevent the deterioration of cardiac function, but the effect is relatively slight. The patient accepted a heart transplantation at the age of 60. Two younger sisters of the proband, II8 and II10, did not show significant symptoms of heart dysfunction, except for a slight fatigue for II10. Still, the ECG showed a similar low potential in all leads, the T waves in II, III, AVF, V4–V6 are inversed in II8 and biphasic in II10. The PVCs of II10 is 296 times/24 h, with half of them (149 times) are multifocal. As for II8, only 8 PVCs and 11 APC were observed in 24 h. The echocardiography also showed different severity between the two individuals. The left ventricle of II8 showed a slight enlargement and a normal ejection function (LVEDD 56 mm, LVEF 55.5%), while II10 has larger ventricular volume and less ejection function (LVEDD 65 mm, LVEF 40.8%). As for the descendants at their age of 30 s, no one has a clinical symptom to date. However, the two PLN-14Rdel carriers showed abnormal changes in heart like their mothers. III5 nearly fulfils the diagnosis of DCM (LVEDD 59 mm, EF 52.6), and a regional wall motion abnormality was observed. For III6, the LV structure and function were relatively normal (LVEDD 51 mm, LVEF 55.3%) considering her overweight (BMI 35.2). for III5, no special was found in ECG and Holter except for some abnormal flat T waves in II/III/AVF, the R wave in III and V6 is relatively low but of no significance.

### Cardiac magnetic resonance: LGE pattern and ECV

**Figure 3:**
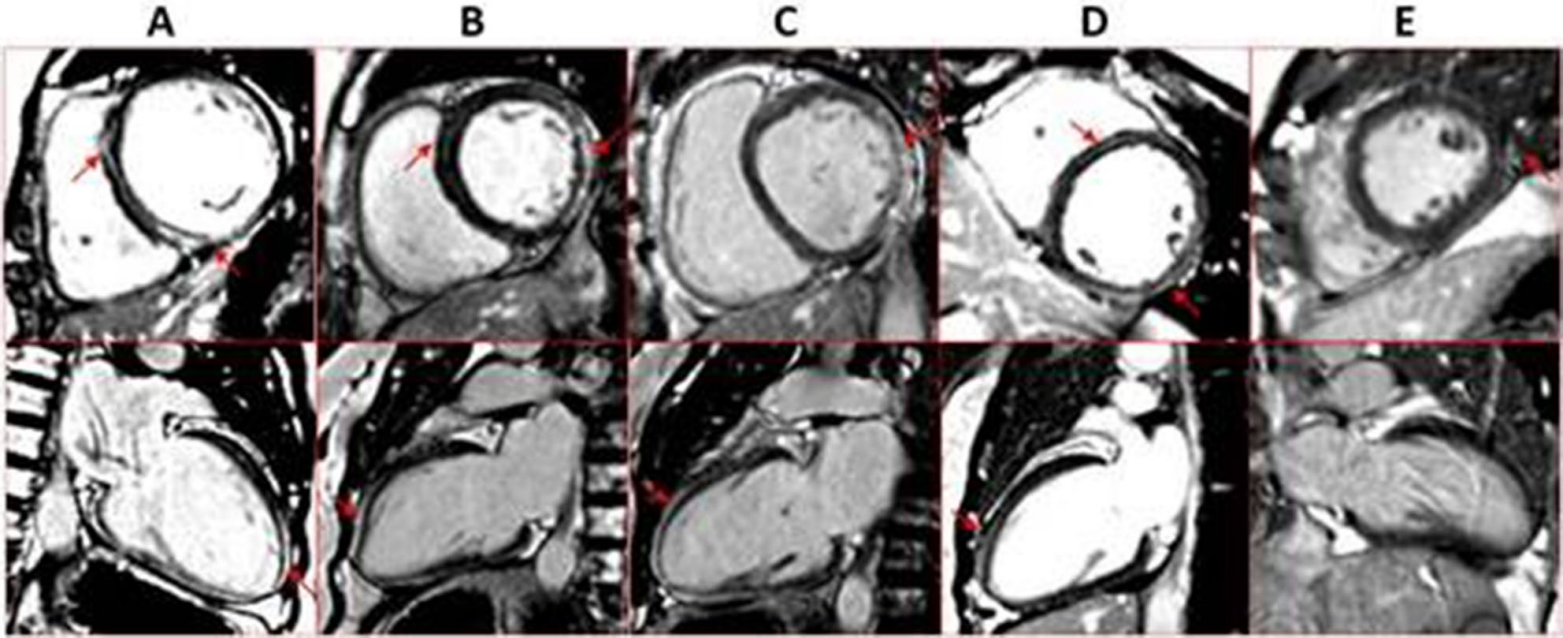
CMR images of II6(A), II8(B), II10(C), III5(D), III6(E), arrow shows the most significant late gadolinium enhancement (LGE). Except for III6(E), All of the members showed significant LGE at the lateral wall of LV and linear LGE at the midmyocardium, especially at the intraventricular septum, in at least two axis views. III6(E) showed a suspicious enhancement at the left lateral wall at the short axis view and no linear LGE at the midmyocardium.

For the proband II6, LGE was significant in the sub-epicardial layer of LV lateral wall, and linear mid-wall enhancement in the septum from basal to apical level. Both of the two sisters (II8 and II10) showed significant sub-epicardial LGE in the LV lateral wall, and linear mid-wall on septal myocardium. III5 also has a significant LGE which spreads over the lateral wall of LV, but the mid-wall LGE is relatively slight. As for III6, the LGE was slight but visible in part of the posterior-lateral wall of LV at basal level. The LGE images were shown in Fig. 3. In addition, the examination of T1mapping and ECV of those members, suggested remarkable diffuse fibrosis indicated by significantly increased native T1 and ECV. For instance, the patients with obviously global left ventricular involvement and clinical symptoms have the highest ECV (43.2% for II10 and 42.4% for II6, respectively). Those showing no symptoms but a significant posterior lateral wall LGE have middle level of ECV (34.9% for II8, 32.4% for III5). As for III6, the morphology and function of heart is normal and the ECV is also within the normal range (24.5%) (Table 1).

**Table 1:** Clinical baseline and cardiac magnetic resonance imaging characteristics of PLN-R14del carriers.

Subject	Age	Sex	Symptoms	ECG	LVEDD	LVEF	T1M	T2M	ECV	LGE pattern
II6	60	F	Chest tightness upon activity, shortness of breath	Low amplitude of QRS in all leads; abnormal T waves in V2-V6; complete right bundle branch block	6.9	40	1354	45	42.4	Intraventricular septum linear enhancement, intraventricular septum patchy enhancement, left lateral wall epicardium enhancement
II8	57	F	None	Low amplitude of QRS in all leads; inversed T waves in II, III, AVF, and V4–V6	5.6	55.5	1324	44.3	34.9	Slight intraventricular septum enhancement, left lateral wall epicardium enhancement
II10	54	F	Fatigue	Low amplitude of QRS in all leads; biphasic T waves in II, III, avF, and V4–V6	6.5	40.8	1375	45.6	43.2	Slight intraventricular septum enhancement, left lateral wall epicardium enhancement
III5	32	F	None	Flat T waves in II, III, and AVF	5.9	52.6	1249	46.1	32.4	Slight intraventricular linear enhancement, left lateral wall epicardium enhancement
III6	31	F	None	Normal	5.1	55.3	1230	42.2	24.5	Suspicious left lateral wall epicardium enhancement

## Discussion

PLN plays an important role in the complex and dynamic regulation of diastole intracellular calcium. The flexibility of its inhibition ability relies on the upstream regulation from PKA and the proportion of functional PLN forms inside cardiomyocytes. In this context, whether a loss or gain of PLN function caused by a pathological mutation has the potential to induce disorder of intracellular calcium.

Mutations in PLN cause cardiomyopathy in a distinct way, as they may not directly influence the structural or functional characteristics of cardiomyocytes, but disrupt the calcium homeostasis inside cardiomyocytes and interrupt the rhythm of myocardial contraction. The known mutations in human include R14del, R9C, R9L, R9H, Leu-39stop, and R25C. Each mutation has a unique mechanism to eventually induce cardiomyopathy. In brief, the presumed mechanisms are: (1) Completely or partially losing the function to inhibit SERCA; (2) Interfering the normal PLN to inhibit SERCA in a dominant negative way. (3) Failing to be phosphorylated and regulated by PKA, or further, disabling the normal function of PKA^6,10,11^. For instance, R9C is an Arg-Cys mutation which occurs in the cytoplasmic region (1–16). This region is critical for PKA to recognize and bind PLN. Therefore, the mutated PLN blunts the effect of β-adrenergic stimulation. As the R9C-PLN lost most of its function as PLN, the consequence is uncontrolled SERCAs, which permanently enhance the calcium reuptake and sparks, intensify oxidative stress, and ultimately cause the loss of contraction function of cardiomyocytes^6,12^.

R14del is a well-known PLN mutation in Dutch people, with 10–15% of both dilated cardiomyopathy and arrhythmogenic cardiomyopathy patients are claimed to be caused by PLN-R14del^13^. Unlike R9C, R14del-PLN retains partial function of normal PLN. However, the complex formed with SERCA cannot be phosphorylated by PKA, thus preventing normal PLN to bind SERCA, ending with a super-inhibition of SERCA^4,6^. During the past decade, a few specific clinical characteristics related to this kind of patients have been observed, including a female dominant morbidity^14^, late-onset heart failure symptoms together with early-onset sudden death risk^13^, a low voltage of QRS and abnormal T wave mainly on anterior-lateral precordial leads^5^, and an involvement in right ventricular and anterolateral wall of left ventricular^8^. Few studies were made to illuminate the underlying mechanism of this genotype–phenotype relationship. It is assumed that improper systole-diastole rhythm caused by disordered intracellular calcium activity prefers to impact the lateral of ventricular which is more sensitive to wall strain^8^, and ECG reveals the pathological region to some extent^7^.

The frequency of PLN-R14del is relatively low outside Dutch, especially for Asians. To the best of our knowledge, no R14del family was reported in China, although some researches did try to investigate PLN mutations in Chinese population^15,16^. In this study, we discovered a PLN-R14del family existing in Southwestern China, of which 5 members carrying this mutation share similar clinical characteristics as reported. ECG showed a low QRS potential and abnormal T wave especially in precordial leads. Holter caught moderate to severe ventricular arrythmias in patients at their age of 50 s, supporting the past studies. These characteristics are not apparent for younger non-symptomatic carriers at their age of 30 s. On the other hand, CMR successfully recognized the early myocardium change which is mainly distributed on the post-lateral wall of left ventricular. We describe the typical pattern of LGE in PLN-R14del patients as a mild linear enhancement in midmyocardium and a significant diffused enhancement in lateral wall of LV. Post-lateral wall epicardium LGE seems to be the earliest pathologic change among R14del carriers, which underlies the essential role of CMR to screen out potential early-phase inherited cardiomyopathy patients. We assume that inhibited SERCA function and failed PKA regulation caused by PLN-R14del first impact the post-lateral wall because of its sensitivity to wall stress. As the fibrosis extends, re-entry circuits are formed to cause frequent ventricular arrythmias and accelerate the dysfunction of whole left ventricular. ECV also showed remarkable diffuse fibrosis in PLN-R14del carriers. For the two symptomatic and confirmed DCM patients, the mean ECV value was 42.8%, which was significantly higher than the average level in our idiopathic DCM study (31.1 ± 5.0%, data not shown) and other DCM researches^17,18^. Those findings implicate a higher diffuse myocardial fibrosis level in PLN-mutated patients, and that ECV is an efficient means for the quantitation and monitoring of myocardial fibrosis changes. In addition, we had observed that the ejection function of LV can maintain normal or mildly reduced (> 40%) for a long period before surgery treatment is needed, implicating that PLN-induced DCM is a chronic progress and cardiac contraction efficiency is not the first impacted. In this study, we didn’t find any extra right ventricular involvement even in the biopsy of the proband (data not shown), though it has been reported in previous studies^8,19^. The ARVC and DCM forms of PLN-R14del induced cardiomyopathy seem to have different mechanism despite the same genetic causes and need to be treated distinctively. As all of our carriers are female, it’s hard to conclude the gender difference between male and female. Still, the high penetrance of this mutation was so obvious that each female carrier identified should be carefully evaluated at the early age.

Different genotypes lead to different phenotypes, and CMR is an efficient way to distinguish the difference. Classical sarcomere mutations like MYH7, TTN induce linear LGE mainly in the intraventricular septum, whereas PLN and desmosomal mutations impact more on the epicardium of the lateral wall^8^. Although increasing evidence have shown that PLN-induced cardiomyopathy may have a distinct pathogenesis comparing to those caused by classical sarcomere mutations, the underlying mechanisms are not fully explained, nor did a comprehensive guideline come out to effectively identify early-stage patients and prevent the high prevalence of lethal arrhythmia^20^. As the PLN-14Rdel blocks β-adrenergic stimulation itself, the routine drugs like β-blockers may exert different effect on this kind of patients. Further study is needed to explore and maximum the effect of early medical intervention on potential patients carrying this kind of mutation.

## Conclusion

We report the first PLN-R14del DCM family in China and its clinical characteristics. ECG change in precordial leads was a common phenomenon among diagnosed patients, and Holter showed mild to severe ventricular arrythmias. The typical pattern of LGE on the post-lateral wall of left ventricular precedes any other pathological changes in young non-symptomatic carriers. ECV revealed severe diffuse myocardial fibrosis in such patients. Our study highlights the importance of CMR for the early discovering and distinguishing of certain genetic-caused cardiomyopathy.

## References

[1] Weintraub RG (2017). Dilated cardiomyopathy. Lancet.

[2] MacLennan DH, Kranias EG (2003). Phospholamban: A crucial regulator of cardiac contractility. Nat. Rev. Mol. Cell Biol..

[3] Nelson SED (2018). Effects of the Arg9Cys and Arg25Cys mutations on phospholamban's conformational equilibrium in membrane bilayers. Biochim. Biophys. Acta Biomembr..

[4] Young HS (2015). Deception in simplicity: Hereditary phospholamban mutations in dilated cardiomyopathy. Biochem. Cell Biol..

[5] Haghighi K (2006). A mutation in the human phospholamban gene, deleting arginine 14, results in lethal, hereditary cardiomyopathy. Proc. Natl. Acad. Sci. USA.

[6] Ceholski DK (2012). Lethal, hereditary mutants of phospholamban elude phosphorylation by protein kinase A. J. Biol. Chem..

[7] Posch MG (2009). Genetic deletion of arginine 14 in phospholamban causes dilated cardiomyopathy with attenuated electrocardiographic R amplitudes. Heart Rhythm..

[8] Sepehrkhouy S (2017). Distinct fibrosis pattern in desmosomal and phospholamban mutation carriers in hereditary cardiomyopathies. Heart Rhythm..

[9] Wallis, Y. et al. Practice Guidelines for the Evaluation of Pathogenicity and the Reporting of Sequence Variants in Clinical Molecular Genetics. Association for Clinical Genetic Science (2013).

[10] Abrol N (2015). Acute inotropic and lusitropic effects of cardiomyopathic R9C mutation of phospholamban. J. Biol. Chem..

[11] Ceholski DK (2012). Hydrophobic imbalance in the cytoplasmic domain of phospholamban is a determinant for lethal dilated cardiomyopathy. J. Biol. Chem..

[12] Schmitt JP (2009). Alterations of phospholamban function can exhibit cardiotoxic effects independent of excessive sarcoplasmic reticulum Ca^2+^-ATPase inhibition. Circulation.

[13] van der Zwaag PA (2012). Phospholamban R14del mutation in patients diagnosed with dilated cardiomyopathy or arrhythmogenic right ventricular cardiomyopathy: Evidence supporting the concept of arrhythmogenic cardiomyopathy. Eur. J. Heart Fail..

[14] Kayvanpour E (2017). Genotype-phenotype associations in dilated cardiomyopathy: Meta-analysis on more than 8000 individuals. Clin. Res. Cardiol..

[15] Chen XY (2005). Association between phospholamban gene mutation and dilated cardiomyopathy in the Chengdu area. Sichuan Da Xue Xue Bao Yi Xue Ban.

[16] Zhao CX (2004). Association between mutation of phospholamban gene and dilated cardiomyopathy. Yi Chuan.

[17] Nakamori, S., Dohi, K., Ishida, M., *et al.* Native T1 mapping and extracellular volume mapping for the assessment of diffuse myocardial fibrosis in dilated cardiomyopathy. JACC Cardiovasc. Imaging, 2017:S1936878X1730400X. 10.1016/j.jcmg.2017.04.006 28624408

[18] Youn JC, Hong YJ, Lee HJ (2017). Contrast-enhanced T1 mapping-based extracellular volume fraction independently predicts clinical outcome in patients with non-ischemic dilated cardiomyopathy: A prospective cohort study. Eur. Radiol..

[19] Gho JM (2014). High resolution systematic digital histological quantification of cardiac fibrosis and adipose tissue in phospholamban p.Arg14del mutation associated cardiomyopathy. PLoS ONE.

[20] Hof IE (2019). Prevalence and cardiac phenotype of patients with a phospholamban mutation. Neth. Heart J..

